# Guided tour of hidden tracts in the pelvis: exploring pelvic fistulas

**DOI:** 10.1007/s00404-021-06144-1

**Published:** 2021-07-20

**Authors:** Iris E Chen, Regan Ferraro, Lucy Chow, Simin Bahrami

**Affiliations:** Ronald Reagan, UCLA Medical Center, 757 Westwood Plaza, Suite 1638, Los Angeles, CA 90095 USA

**Keywords:** Pelvis, Fistula, Tract

## Abstract

**Background:**

Fistulas are an abnormal connection between two or more epithelial surfaces. When fistulization between adjacent structures occurs in the pelvis, there is almost invariably significant associated morbidity and impact on a patient’s quality of life. Imaging may aid in the diagnosis of pelvic fistulas and is essential to identify any associated pathology, define the course of the fistula, and aid in pre-surgical planning.

**Purpose:**

This article aims to review the wide array of clinical and imaging presentations of fistulas in the pelvis, with a focus on the radiologists’ role in managing this challenging entity.

**Methods:**

This article will review each classification type of fistula.

**Results:**

Pelvic fistula is a devastating condition that causes significant morbidity and evaluation can be challenging.

**Conclusions:**

Imaging, and particularly MRI, plays a vital role in the diagnosis, characterizing the course of a fistula and demonstrating associated complications, which are essential to guide treatment decisions.

## Introduction

Fistulas are an abnormal connection between two or more epithelial surfaces. When fistulization between adjacent structures occurs in the pelvis, there is almost invariably significant associated morbidity and impact on a patient’s quality of life. This is exacerbated by the difficulty of diagnosing pelvic fistulas clinically [1]. Imaging may aid in the diagnosis of pelvic fistulas and is essential to identify any associated pathology, define the course of the fistula, and aid in pre-surgical planning. This article aims to review the wide array of clinical and imaging presentations of fistulas in the pelvis, with a focus on the radiologists’ role in managing this challenging entity.

### Classification

Fistulae in the pelvis may be classified by the two structures involved and include genitourinary, intestinogenitourinary and genitocutaneous fistulas. Genitourinary fistulas include vesicouterine, vesicovaginal, urethrovaginal, and ureterovaginal fistulas. Intestinogenitourinary fistulas include colouterine, colovaginal, colovesical, and rectovaginal fistulas. A variety of cutaneous fistulas may form in the pelvis, including genitocutaneous fistulas such as perineovaginal. Additionally, some patients may have a compound or mixed fistulae, involving more than two pelvic organs [2, 3].

### Clinical presentation

Clinical presentation varies by the structures that have fistulized. Genitourinary fistulas may cause incontinence, vaginal urine leakage, localized pain, vaginal pruritis, perineal skin irritation, and recurrent infections of the genitourinary tract. Colovesical and enterovesical fistulas typically produce gas or fecal material in the urine. Changes in vaginal discharge, recurrent vaginal infection or abnormal passage of stool, mucus or flatus per the vagina suggests the presence of a rectouterine, rectovaginal, or colovaginal fistula [2, 4]. Small fistulae may be sometimes present without symptoms.

## Evaluation of fistulas

Radiologic evaluation of fistulas is paramount in their diagnosis. In some cases, imaging may provide direct visualization of a fistulous tract. In other cases, imaging may demonstrate focal wall thickening, the presence of air or contrast material in a non-anatomic location, or loss of a normal soft tissue plane [5].

### Non-radiologic evaluation

Radiologic diagnosis can be complemented by physical and laboratory examinations. In addition to a thorough physical exam, a variety of diagnostic tests can help identify the presence of a fistula. Vaginal fluid analysis can be performed to test for urea, creatinine, and potassium, which would suggest a genitourinary fistula. In a pyridium test, a tampon placed in the vagina is examined for staining after the administration of oral phenazopyridine. If positive, this suggests the presence of a ureterovaginal fistula. This can be performed in tandem with a methylene blue test, where methylene blue is instilled retrograde into the bladder and a tampon in the vagina is examined for the presence of blue dye, indicating a vesicovaginal fistula. When done concurrently, this is referred to as the double-dye test. Cystoscopy should also be performed for direct visualization of genitourinary fistulas [4]. A speculum or half‐speculum can be used to look for red granulated tissue within the vagina if concerned for a colovaginal fistula.

### Radiologic evaluation: modalities

Fluoroscopic evaluation via cystogram, excretory urography, vaginography, water-soluble enemas and fistulograms can be used to evaluate pelvic fistulas. Fluoroscopy is widely accessible and cost-effective. Additionally, real-time imaging on fluoroscopy can be advantageous as the radiologist can utilize different projections and provocative maneuvers to visualize a fistula. Oblique and lateral views can be particularly helpful. However, fluoroscopy alone is somewhat limited in that the radiologist has limited visualization of associated complications, and there is little anatomic detail.

Similar to fluoroscopy, computed tomography (CT) is also broadly available and cost-effective. CT also offers increased sensitivity and accuracy for the detection of fistulas compared with fluoroscopy. Multiphasic acquisition (e.g. unenhanced, contrast enhanced, and delayed or excretory phases) are essential. Even if a fistula is difficult to directly visualize, there may be indirect signs such as gas or contrast in the bladder [6]. Additionally, contrast may be instilled in the bladder, vagina, or gastrointestinal tract depending on the fistula of interest. CT urography and CT cystography are initial imaging modalities to evaluate ureter and bladder fistulae, respectively [1]. Furthermore, if there is a skin sinus tract, contrast can be instilled percutaneously with CT sinogram to determine underlying fistula anatomy. Multiplanar reconstructions offer improved spatial understanding of fistulae and can be particularly helpful to characterize the involved anatomy. The primary limitation of CT is that fistula visualization can be dependent on contrast timing.

MRI is the examination of choice for evaluating pelvic fistulas due to its high intrinsic soft tissue contrast, which allows for direct visualization of the tract [1, 5–7]. Multiplanar image acquisition can help delineate anatomy, particularly if images are acquired in the plane of the suspected fistula. MRI can also demonstrate any associated postsurgical changes, inflammatory changes, abscesses, and the presence and extent of malignancy [1]. These benefits make MRI extremely useful to the referring clinicians and surgeons who will use this information to optimize treatment and surgical planning. Actively inflamed fistulous tracts will demonstrate high signal on T2-weighted images and enhance on T1-weighted post contrast images. Fat‐saturation technique can better delineate T2‐hyperintense fistulae with inflammatory changes, whereas images without fat saturation offer better contrast resolution and anatomic characterization [1]. Abscesses are important to detect to decrease the risk of fistula recurrence and are visualized on post contrast T1‐weighted imaging with fat suppression [8]. Diffusion-weighted imaging can be useful in patients who cannot receive intravenous contrast and will demonstrate diffusion restriction in the setting of active inflammation. Delayed sequences can be added to capture contrast excretion as well. However, motion artifact can greatly degrade image quality, and similar to CT, the contrast timing can impact fistula visualization.

Radiographs and ultrasound offer little diagnostic value in the setting of pelvic fistulae. Radiographs are quick and may be helpful to easily visualize presence of intraperitoneal or intravesical air. Endoanal ultrasound can be used in a conjunction with MRI to evaluate the anal sphincter in anovaginal fistulae [1]. Otherwise, evaluate of pelvic fistulae by ultrasound is limited by overlying bowel gas and poor soft‐tissue contrast.

## Imaging fistulas

Fistulas have numerous causes including malignancy, birth trauma and gynecologic pathologies as well as infectious, inflammatory and iatrogenic etiologies [9].

### Genitourinary fistulas

Genitourinary tract fistulae include any fistula between the female genital tract and the bladder, urethra, or ureters, with vesicovaginal fistulas being the most common. Worldwide, obstetric trauma is the most common cause of genitourinary fistula formation. Pressure necrosis in the setting of obstructed labor often causes large fistulae. In low- and middle-income countries in the Middle East, Africa and Asia, incidence of birth-related fistulas is estimated at one to three out of 1000 deliveries [10]. In the developing world where prenatal care is widespread, fistula from birth trauma itself is significantly less common, but does occur after severe birth-related perineal lacerations or after C-section or other birth-related instrumentation [4].

**Fig. 1:**
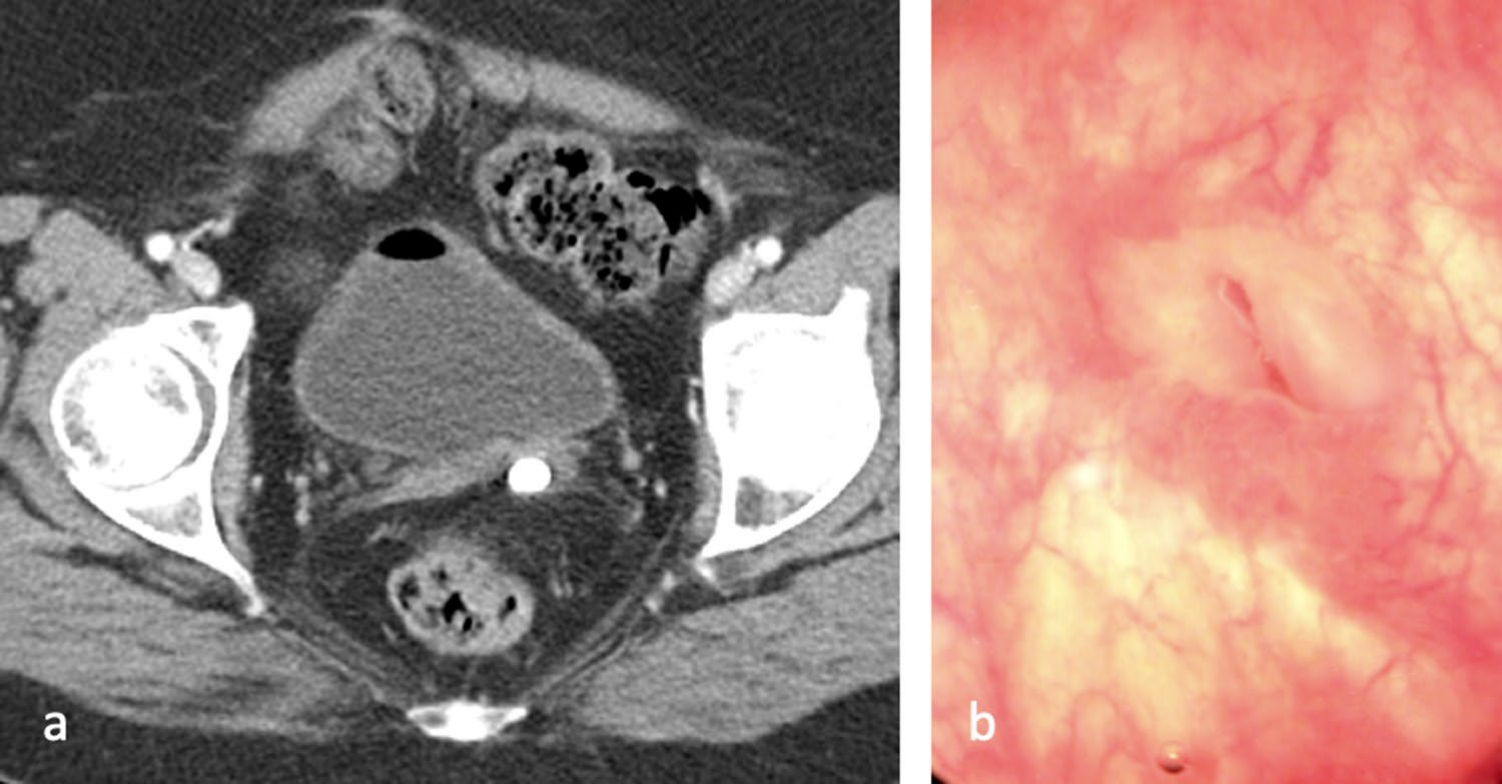
50-year-old female with history of hysterectomy complicated by vesicovaginal fistula status post repair presenting with dysuria. Axial CT through the pelvis **a** demonstrates a bladder stone posterior to the bladder and adjacent to the left vaginal cuff. Cystoscopy **b** demonstrates a defect in the bladder wall related to a sinus tract from the previous vesicovaginal fistula repair. Surgery was later performed and the stone was removed from the bladder sinus tract

Postsurgical causes of pelvic fistulae are more common in developed countries. Postoperative genitourinary fistula typically occurs as an early complication (less than 30 days post-operative) after gynecologic surgery [3]. Incidence of posthysterectomy fistula has been estimated at 0.1%, with vesicovaginal fistula occurring in 1 out of 455 to 1800 hysterectomies [4]. Fistula formation may occur secondary to a variety of intraoperative issues, including bladder injury during forceful blunt dissection of the bladder from the uterus causing a tear or devascularization of the posterior bladder wall, as seen in Fig. 1. Additionally, a vaginal cuff suture errantly incorporated into the bladder wall may cause tissue ischemia and necrosis.

Neoplasm involving any of the pelvic organs (cervix, uterus, vagina, bladder, colon, rectum, prostate) can cause fistula from direct tumor invasion. Approximately 2.5% of patients with gynecologic malignancy develop fistulas, with vesicovaginal and colovaginal being most common [11].

**Fig. 2:**
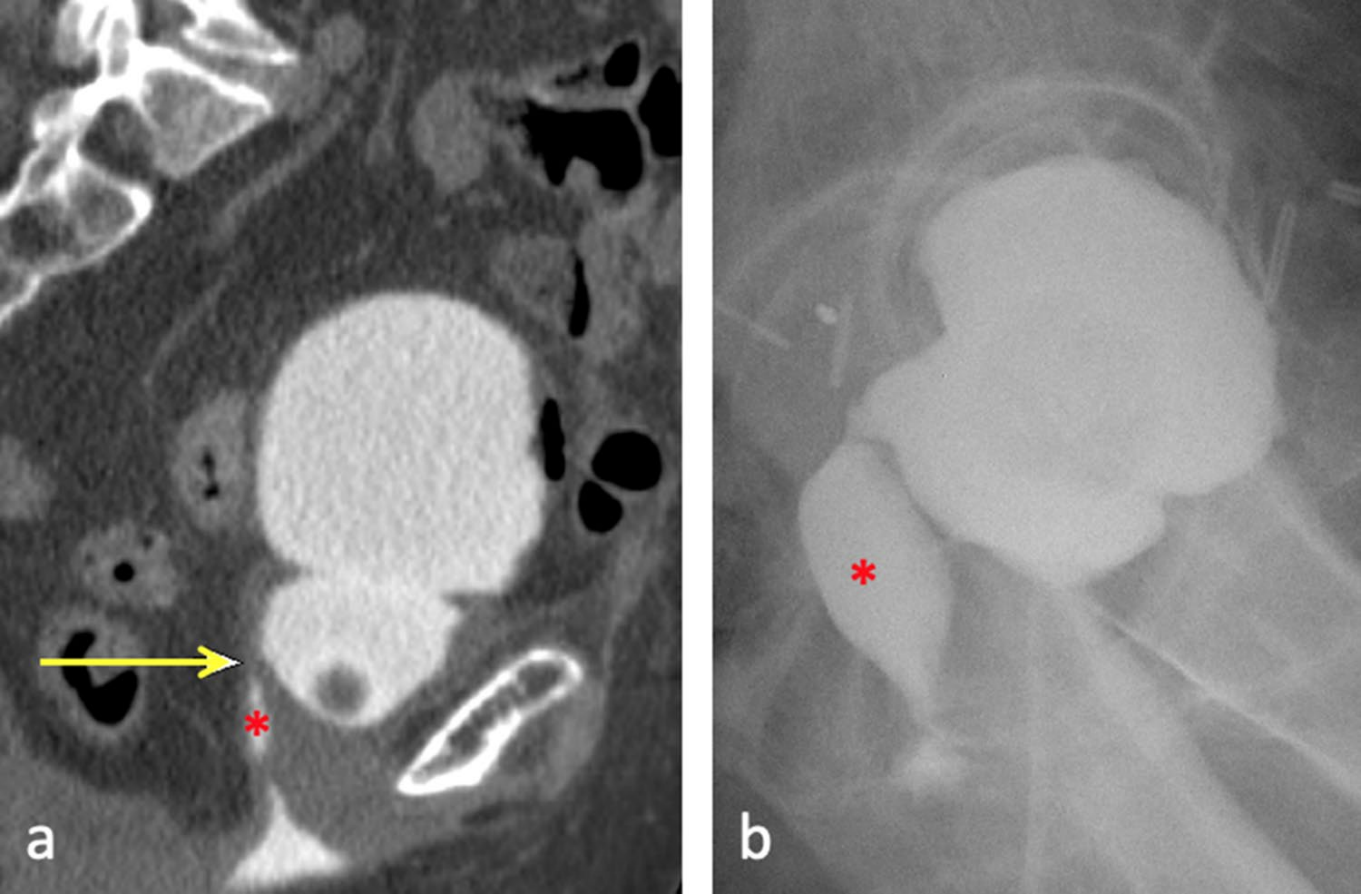
71-year-old female with cervical cancer status post hysterectomy and pelvic radiation. Sagittal CT Cystogram **a** demonstrates contrast in the vagina (*) with a fistulous connection to the bladder (arrow), representing a vesicovaginal fistula. Lateral cystogram **c** demonstrates injection of contrast into the bladder and immediate leakage of contrast through the vagina (*) in the region of the vaginal cuff, compatible with vesicovaginal fistula

Post-radiation therapy fistula formation can be caused by tumor downsizing or fibrosis and tissue necrosis. Fibrosis is a later complication, occurring 1–2 years after treatment after external beam radiation or brachytherapy of the vaginal vault or prostate. Radiation-induced fibrosis and loss of normal anatomy and soft tissue planes causes endarteritis obliterans and ischemic necrosis, allowing fistula formation [6, 12, 13]. There is a reduced incidence now given advances in targeted radiation therapy [6]. Post-radiation therapy vesicovaginal fistula formation is demonstrated in Fig. 2. Fistula formation after radiation therapy may also suggest disease recurrence and subsequent infiltration of adjacent organs.

**Fig. 3:**
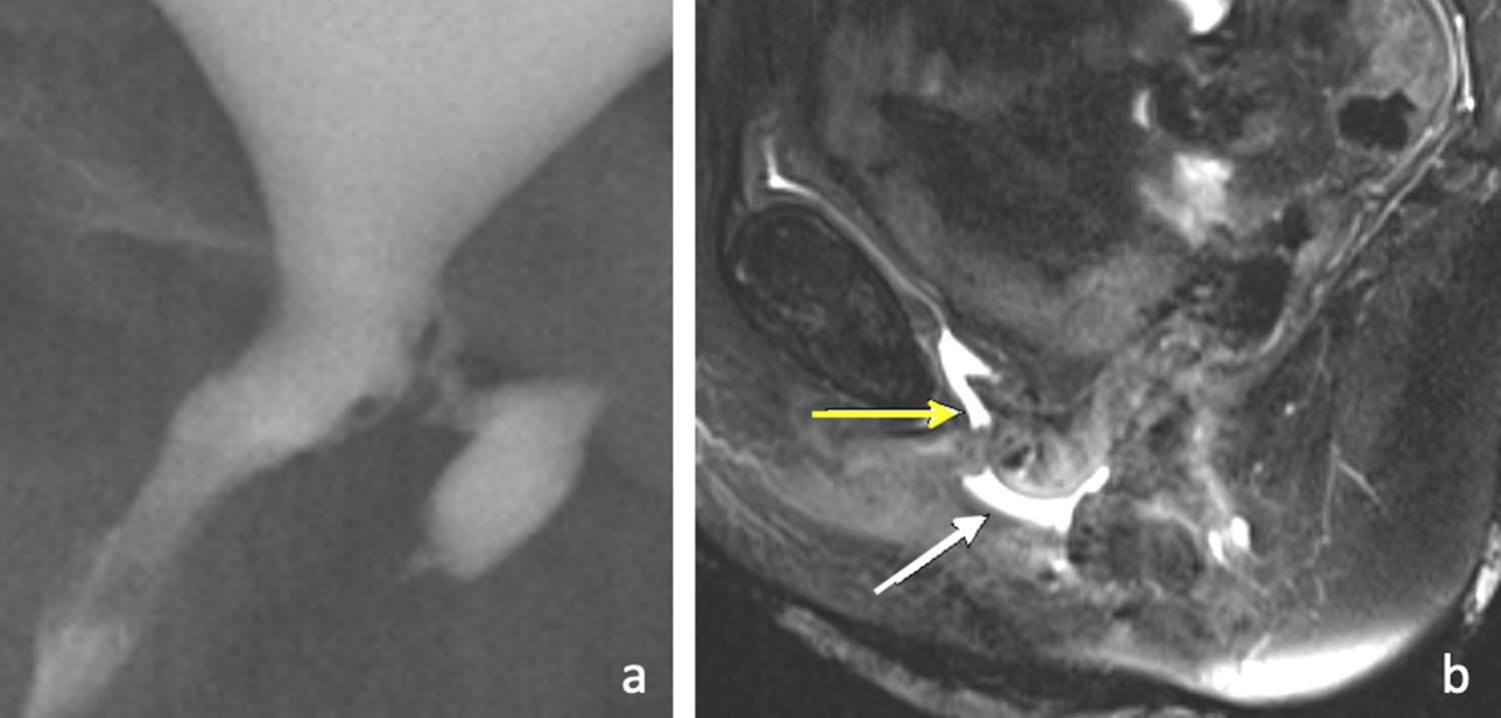
Urethrovaginal fistulas related to pelvic mesh. 47-year-old female with retropubic mesh sling placement complicated by erosion into the urethra visualized by cystoscopy. Subsequent cystogram **a** demonstrates eccentric irregular accumulation of contrast along the left posterior aspect of the bladder neck/proximal urethra in the region of the vagina, with a thin sinus tract compatible with a urethrovaginal fistula. 51-year-old female with history of periurethral and perivaginal mesh excision. Sagittal T2-weighted image **b** demonstrates a thin tract of hyperintense fluid extending down the patulous urethra (yellow arrow). A small collection of T2 hyperintense fluid in the lower vagina (white arrow) is suggestive of a urethrovaginal fistula

**Fig. 4:**
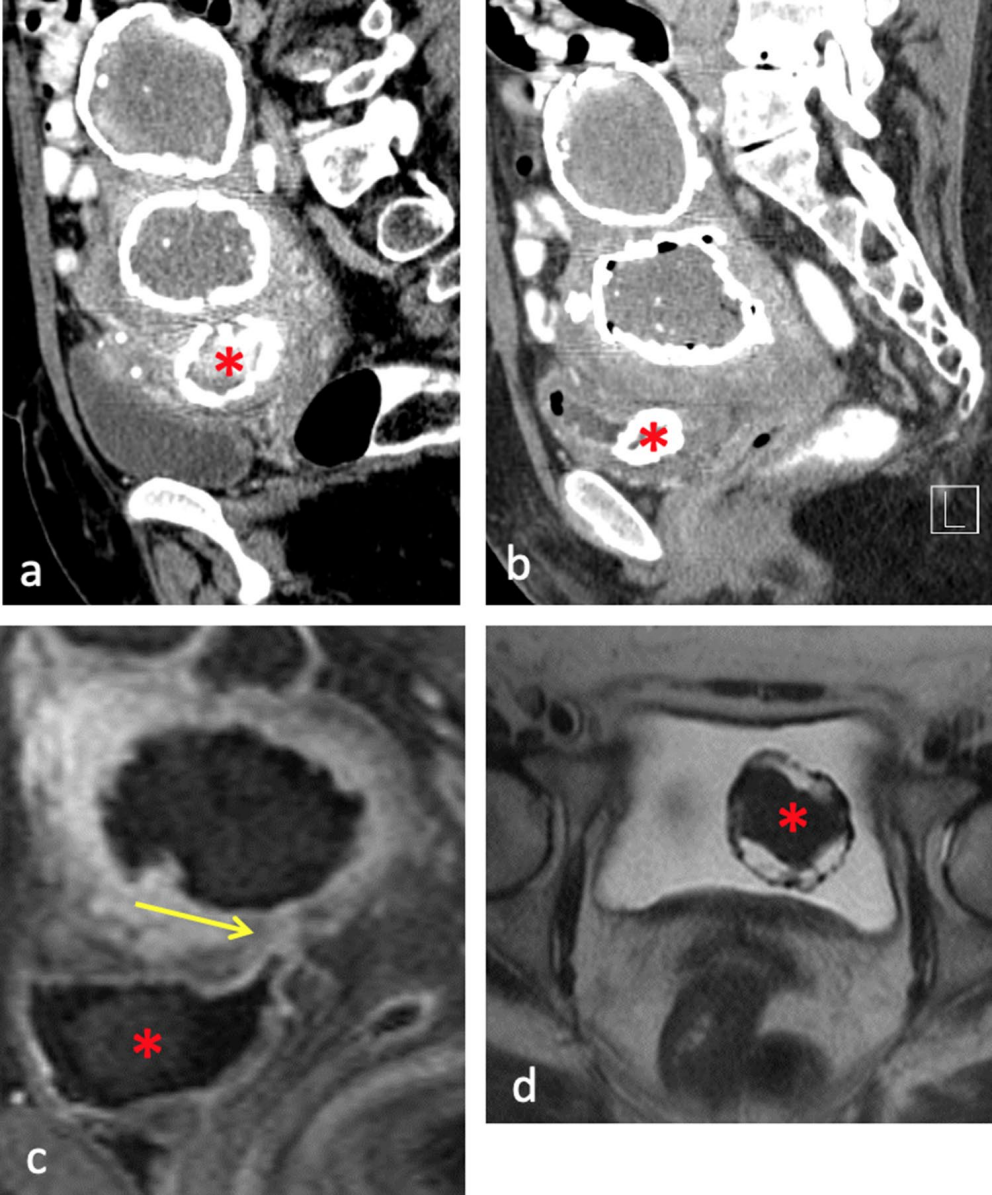
64-year-old female with known calcified uterine fibroids **a** developed dysuria. Repeat CE-CT **b** shows interval erosion of the fibroid (*) into the bladder with wall thickening, mucosal enhancement and foci of air. Post-contrast T1-weighted **c** and T2-weighted (**d**) images confirm fibroid erosion into the bladder and fistulous connection between the uterus and bladder dome (arrow), compatible with vesicouterine fistula. Patient underwent fibroid removal, bladder reconstruction and hysterectomy

Any inflammatory or infectious process in the pelvis may predispose to fistula formation. Intrapelvic foreign body erosion can result in genitourinary fistula, such as erosion of a pelvic mesh into the urethra as illustrated in Fig. 3. Similarly, erosion of a uterine fibroid into surrounding organs may also result in fistulization from direct compression causing tissue necrosis. This etiology should particularly be considered in the setting of an enlarged prolapsing fibroid [14]. Figure 4 demonstrates fibroid erosion into the bladder and subsequent creation of a connection between the uterus and bladder dome, compatible with vesicouterine fistula.

### Intestinogenitourinary fistulas

**Fig. 5:**
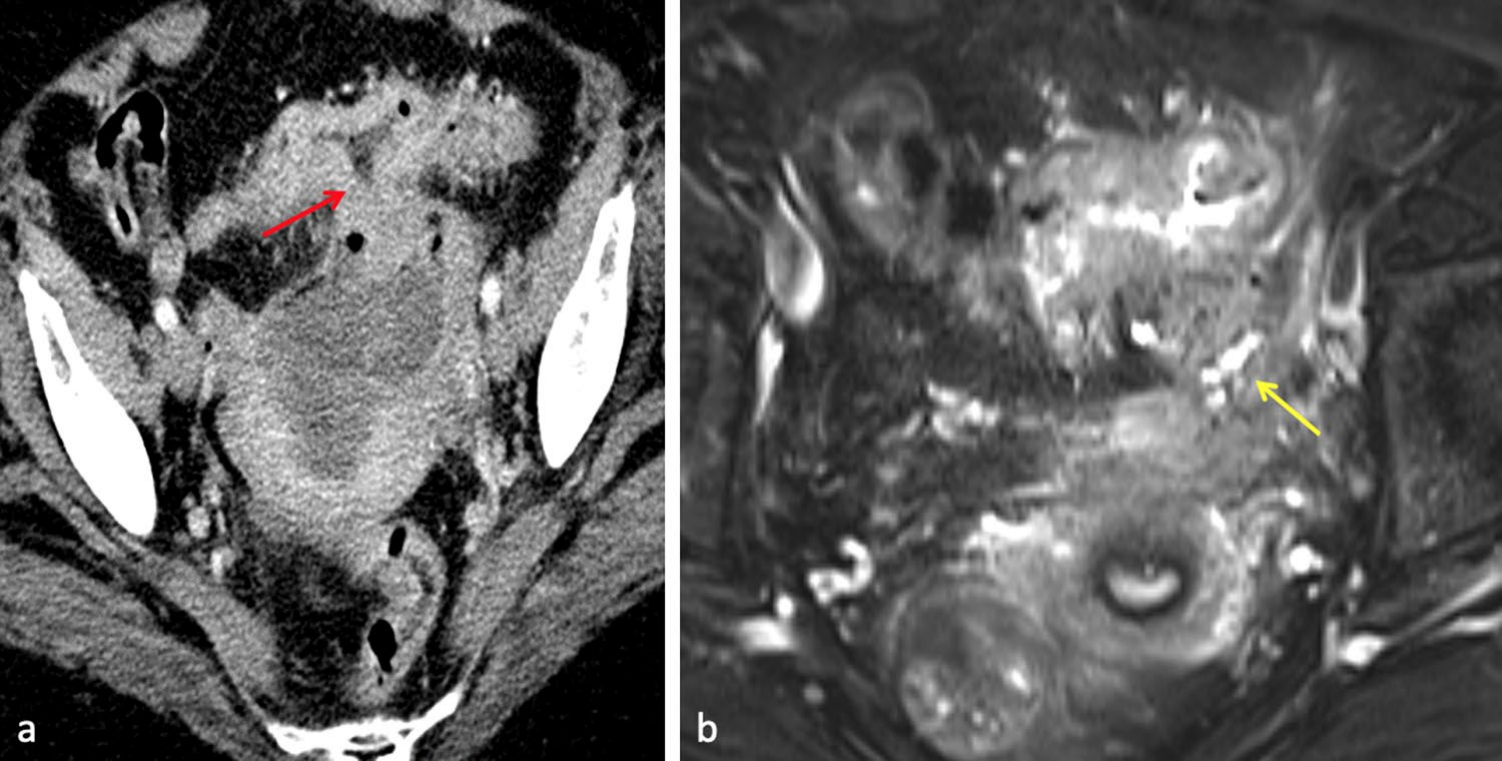
61-year-old female presenting with abdominal pain, fevers, and feculent vaginal drainage. Contrast-enhanced CT **a** demonstrates sigmoid wall thickening and adjacent stranding consistent with acute diverticulitis. Associated abscess and soft tissue tract with locules of gas between the inflamed sigmoid colon and uterine fundus (red arrow) is concerning for colouterine fistula. Axial T2-weighted fat saturated MR **b** demonstrates a hyperintense fistulous tract (yellow arrow). The patient improved with IV antibiotics

Inflammatory processes, in particular diverticular disease and inflammatory bowel disease, commonly cause intestinogenitourinary fistulas. Fistula formation has been reported at a rate up to 14% following acute diverticulitis [15]. Colovesical fistulas are the most common spontaneous fistula to form in the setting of diverticular disease, although other intestinogenitourinary fistulas can also occur, including colouterine fistula as seen in Fig. 5. Colovaginal fistulae secondary to sigmoid diverticulitis most often involve the upper third of the vagina and is caused by direct spread of infection or inflammation, particularly following hysterectomy. Colocutaenous and coloenteric fistulas are more rarely seen compared to other types of fistulas secondary to complicated diverticulitis.

The pathophysiology of fistula formation in Crohn’s disease is not well understood; however, theories include mucosal and transmural inflammation and luminal bacteria [16]. In Crohn’s disease, enterovesical fistulas are most common [17, 18]. MRI is superior in imaging fistulas secondary to inflammatory bowel disease as the modality allows for characterization of complex fistula with multiple tracks, as is often seen in Crohn’s disease. MRI has also been described as a more accurate measure of disease activity given a delayed radiological resolution even after clinical improvement following therapy [19].

Similar to genitourinary tract fistulae, anovaginal and rectovaginal fistulas are most commonly caused by obstetric trauma due to pressure necrosis from prolonged labor. Infections, particularly from chronic granulomatous disease such as tuberculosis, schistosomiasis, and actinomycosis, as well as retained foreign bodies are also associated with intestinogenitourinary fistula formation.

**Fig. 6:**
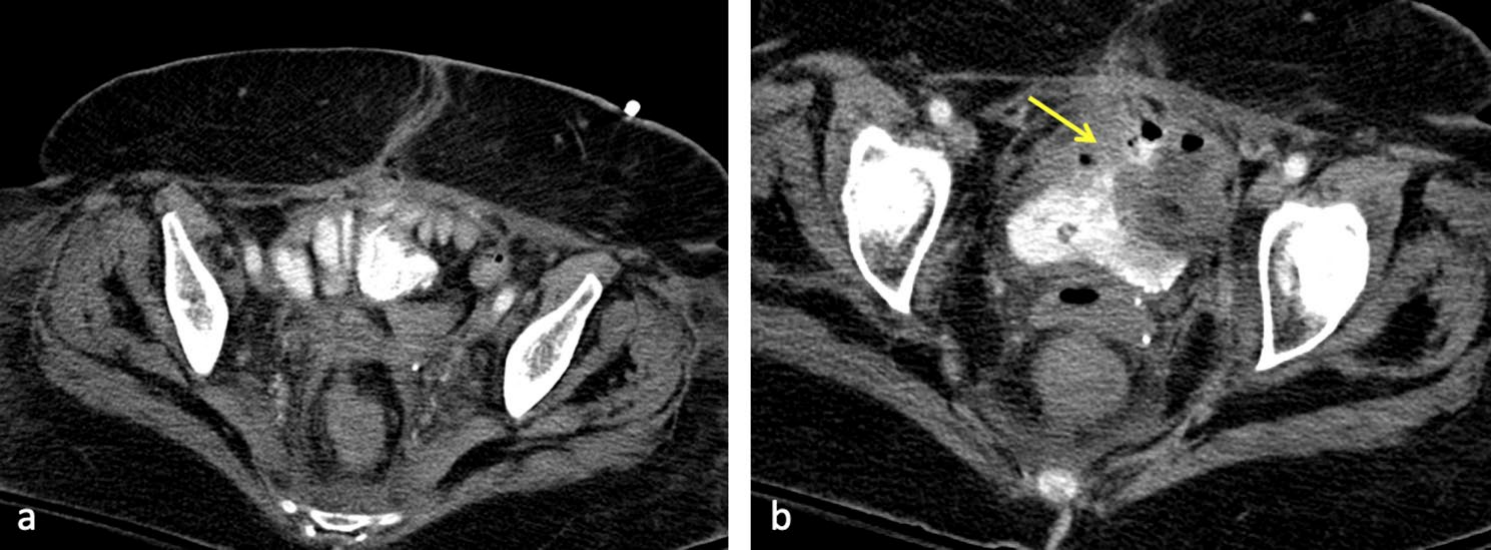
66-year-old female with rectal cancer status post chemoradiation presented with small bowel obstruction. CT of the abdomen and pelvis with oral contrast **a** demonstrates small bowel superior and ventral to the bladder with surrounding inflammatory changes. An air-filled tract extends from small bowel loops to the bladder (**b**, yellow arrow). Air and dense contrast in the bladder is enteric in origin

**Fig. 7:**
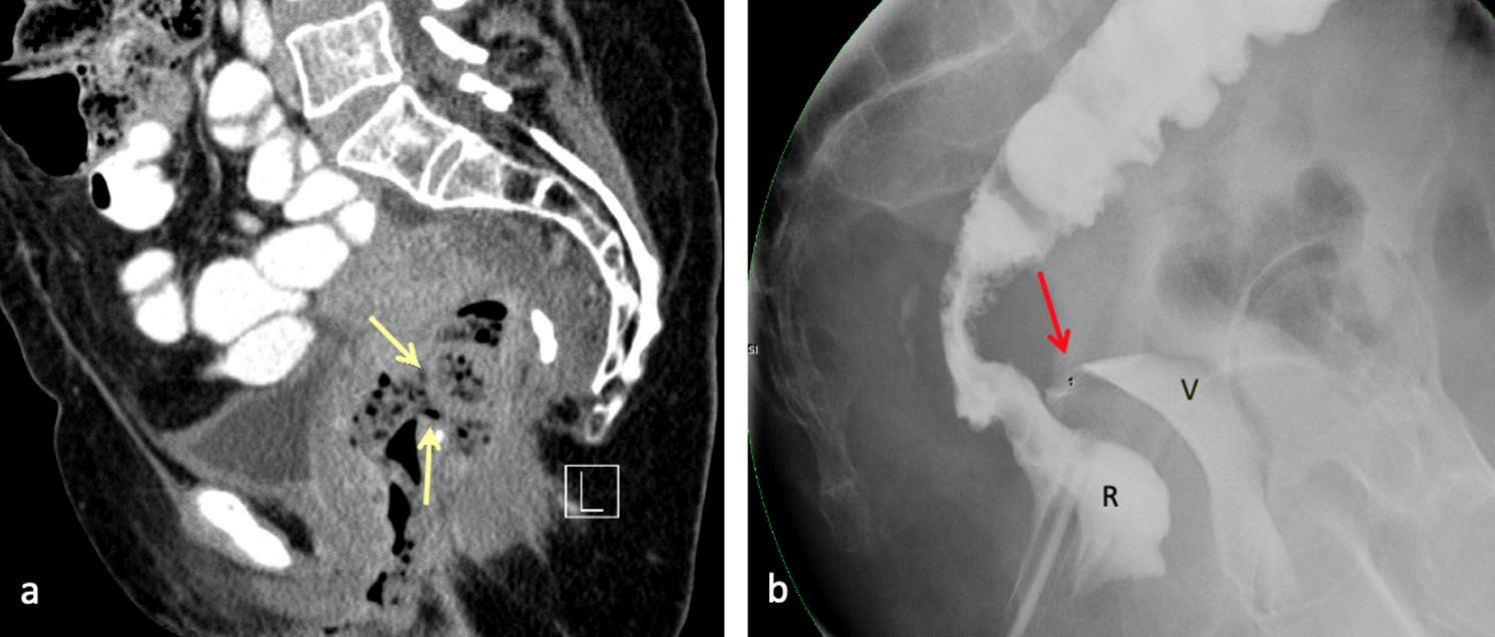
61-year-old female with history of colon cancer status post-radiation and low anterior resection, with subsequent development of radiation-induced rectovaginal fistula. Sagittal contrast-enhanced CT **a** demonstrates direct communication of the lower rectum anteriorly with the vaginal canal (yellow arrows). Feculent material and air are seen within the vaginal canal and there is loss of the normal fat plane between the vagina and rectum, compatible with a rectovaginal fistula. Gastrograffin enema **b** demonstrates contrast material filling the rectum (R) and leaking into the vagina (V) through a narrow fistula (red arrow) extending from the upper rectum near the rectosigmoid junction to the vaginal fornix, consistent with rectovaginal fistula

**Fig. 8:**
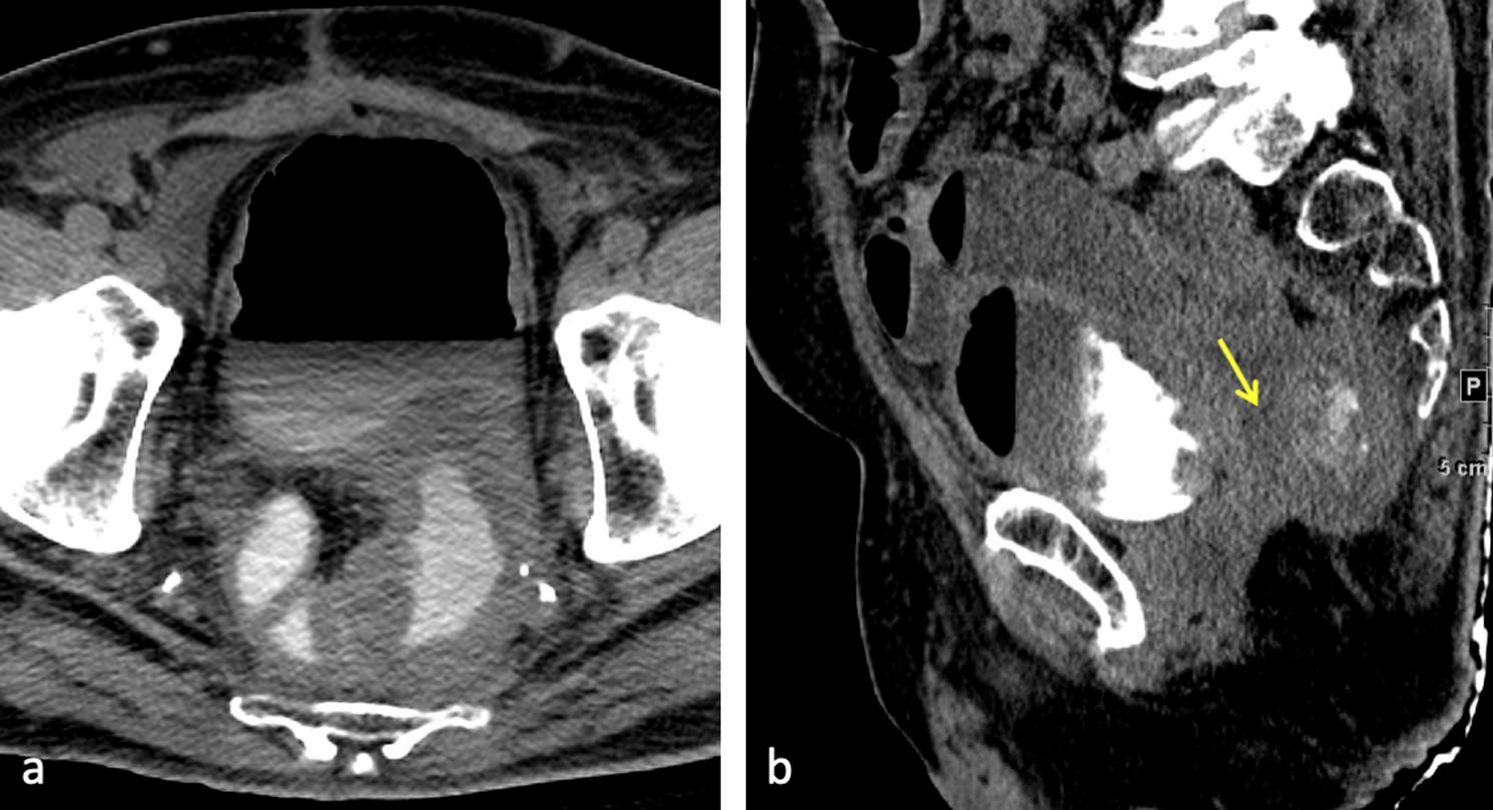
82-year-old male with history of prostate cancer and rectal cancer status post abdominoperineal resection. Axial **a** and sagittal **b** contrast-enhanced CT cystogram demonstrates contrast in the rectum with a fistulous connection to the air-filled bladder (arrow), compatible with a rectovesical fistula

Finally, intestinogenitourinary fistulas can result from pelvic malignancy, either by direct tumor invasion, due to post-radiation therapy, or as a postsurgical complication. Figure 6 demonstrates a colovesical fistula due to post-radiation changes. Similarly, a radiation-induced rectovaginal fistula is shown in Fig. 7. A rectovesical fistula following abdominoperineal resection is depicted in Fig. 8.

### Multiple complex fistulas

**Fig. 9:**
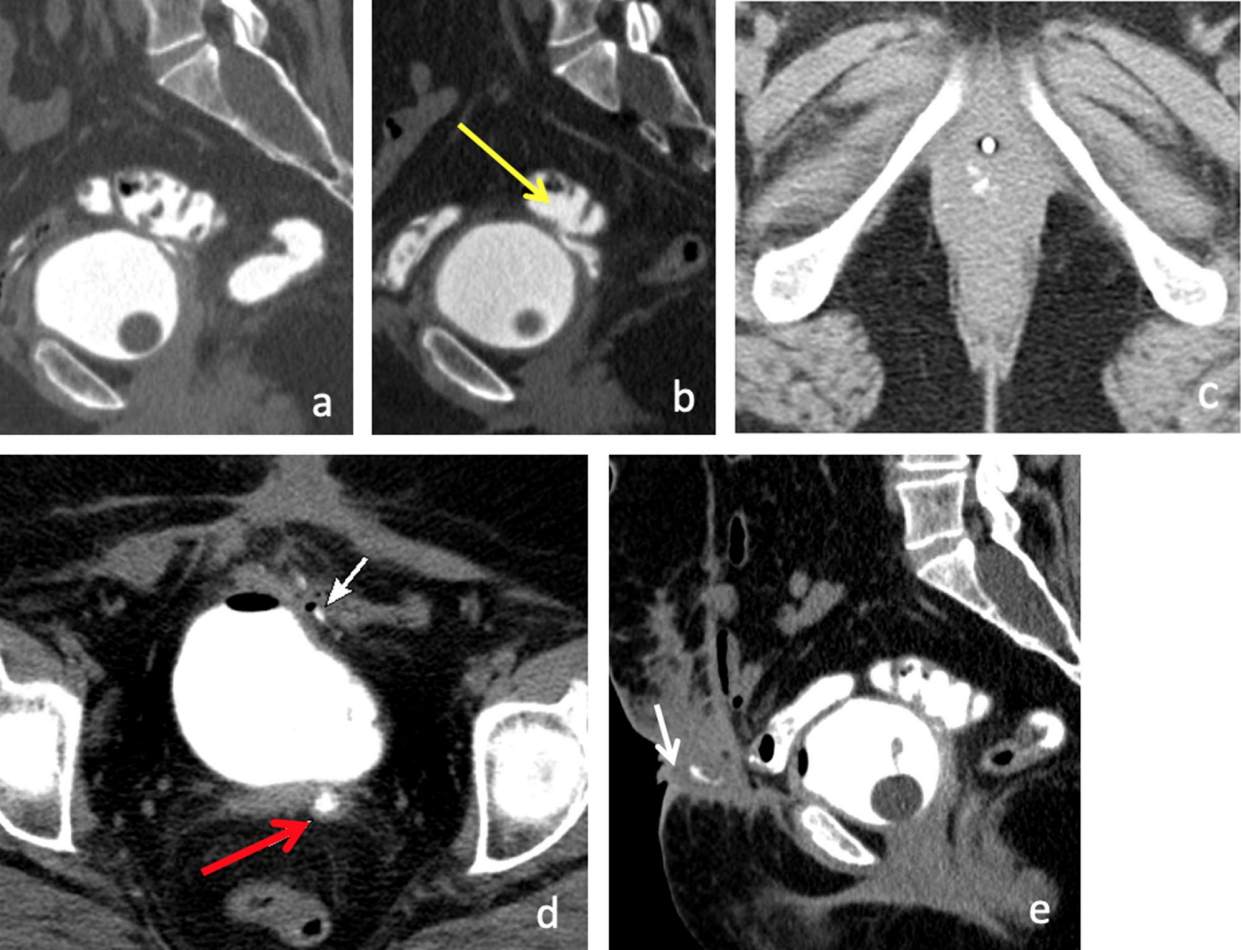
50-year-old female with history of vesicovaginal fistula after hysterectomy. CT cystogram **a** demonstrated opacification of the bladder and rectosigmoid colon, suggestive of a colovesical fistula located at the level of the vaginal apex. Contrast extravasation from the anterior aspect of the sigmoid colon to the vaginal apex (**b**, yellow arrow) is compatible with a colovaginal fistula. Additionally, a small amount of contrast is identified within the introitus of the vagina (**c**). Large bowel is closely adherent to the anterior pelvic wall with adjacent increased soft tissue thickening, foci of gas and contrast in the low anterior pelvic wall (**d**, **e**). These findings are suggestive of a colocutaneous fistula (white arrow). Contrast also seen in vagina (red arrow)

Occasionally, multiple pelvic fistulas may be present simultaneously. Multiple complex fistulas often occur in the setting of prior surgery due to postsurgical changes and active inflammatory processes. An example of multiple complex fistulas is demonstrated in Fig. 9.

## Management

Pelvic fistulas are often difficult to treat, and a multidisciplinary approach with surgery, urology, gynecology, and gastroenterology is frequently necessary for appropriate management [20].

### Conservative management

Conservative management of pelvic fistulas may be appropriate if the fistulous tract is simple, small, and unrelated to malignancy or radiation therapy. Treatment typically involves antibiotic therapy and abscess drainage if present. For fistulas involving the urinary tract, urinary diversion with transurethral catheters, suprapubic catheters or nephrostomy may allow a tract to heal [11, 21]. Sitz baths may be used for symptomatic relief if appropriate. Estrogen therapy can improve tissue revascularization in postmenopausal patients.

### Operative management

The majority of pelvic fistulas require operative management and depends on location, etiology, fistula morphology, and history of prior repair. The edges of a fistula may be surgically debrided or the tract may be excised and closed with sutures [11]. Grafts and flaps (e.g., gracilis flap and Martius flap) may be utilized in the surgical approach which can help promote healing by interposing additional healthy tissue and increasing blood supply to the area [4]. This neovascularization and promotion of granulation tissue may be particularly helpful in recurrent fistulas where there is often a lot of scarring and fibrotic tissue. High fistulas may need to be repaired transabdominally, while low fistulas can be repaired in other ways, including anal, perineal, or vaginal approaches [22].

## Conclusion

Pelvic fistula is a devastating condition that causes significant morbidity and evaluation can be challenging. Imaging, and particularly MRI, plays a vital role in the diagnosis, characterizing the course of a fistula and demonstrating associated complications, which are essential to guide treatment decisions. A multidisciplinary approach is often necessary for adequate treatment.

## Data Availability

Not applicable.

## References

[1] Hyde BJ, Byrnes JN, Occhino JA, Sheedy SP, VanBuren WM (2018). MRI review of female pelvic fistulizing disease. J Magn Reson Imaging.

[2] Yu NC, Raman SS, Patel M, Barbaric Z (2004). Fistulas of the genitourinary tract: a radiologic review. Radiographics.

[3] Paspulati RM, Dalal TA (2010). Imaging of complications following gynecologic surgery. Radiographics.

[4] Rogers RG, Jeppson PC (2016). Current diagnosis and management of pelvic fistulae in women. Obstet Gynecol.

[5] Addley HC, Vargas HA, Moyle PL, Crawford R, Sala E (2010). Pelvic imaging following chemotherapy and radiation therapy for gynecologic malignancies. Radiographics.

[6] Papadopoulou I, Stewart V, Barwick TD (2016). Post-radiation therapy imaging appearances in cervical carcinoma. Radiographics.

[7] Outwater E, Schiebler ML (1993). Pelvic fistulas: findings on MR images. AJR Am J Roentgenol.

[8] Erlichman DB, Kanmaniraja D, Kobi M, Chernyak V (2019). MRI anatomy and pathology of the anal canal. J Magn Reson Imaging.

[9] Moon SG, Kim SH, Lee HJ, Moon MH, Myung JS (2001). Pelvic fistulas complicating pelvic surgery or diseases: spectrum of imaging findings. Korean J Radiol.

[10] Lee JK, Stein SL (2010). Radiographic and endoscopic diagnosis and treatment of enterocutaneous fistulas. Clin Colon Rectal Surg.

[11] Avritscher R, Madoff DC, Ramirez PT (2004). Fistulas of the lower urinary tract: percutaneous approaches for the management of a difficult clinical entity. Radiographics.

[12] Debas HT, Donkor P, Gawande A, Jamison DT, Kruk ME, Mock CN, editors. Essential Surgery: Disease Control Priorities, Third Edition (Volume 1). Washington (DC): The International Bank for Reconstruction and Development / The World Bank; 2015 Apr 2. PMID: 26740991 26740991

[13] Viswanathan AN, Lee LJ, Eswara JR (2014). Complications of pelvic radiation in patients treated for gynecologic malignancies. Cancer.

[14] Nkwabong E, Fomulu JN (2017). Urethrovaginal fistula following vaginal prolapse of a pedunculated uterine myoma: a case report. J Med Case Rep.

[15] Sessa B, Galluzzo M, Ianniello S, Pinto A, Trinci M, Miele V (2016). Acute Perforated Diverticulitis: Assessment With Multidetector Computed Tomography. Semin Ultrasound CT MR.

[16] Lee MJ, Parker CE, Taylor SR (2018). Efficacy of Medical Therapies for Fistulizing Crohn's Disease: Systematic Review and Meta-analysis. Clin Gastroenterol Hepatol.

[17] Badic B, Leroux G, Thereaux J (2017). Colovesical fistula complicating diverticular disease: a 14-year experience. Surg Laparosc Endosc Percutan Tech.

[18] Wade G, Zaslau S, Jansen R (2014). A review of urinary fistulae in Crohn's disease. Can J Urol.

[19] Lee T, Yong E, Ding NS (2020). Radiological outcomes in perianal fistulizing Crohn’s disease: a systematic review and meta-analysis. JGH Open.

[20] Børseth KF, Acharya G, Kiserud T, Trovik J (2019). Incidence of gynecological fistula and its surgical treatment: a national registry-based study. Acta Obstet Gynecol Scand.

[21] Titton RL, Gervais DA, Hahn PF, Harisinghani MG, Arellano RS, Mueller PR (2003). Urine leaks and urinomas: diagnosis and imaging-guided intervention. Radiographics.

[22] Kamiński JP, Tat C, Fleshner PR, Zaghiyan K (2018). Martius flap for persistent, complex rectovaginal fistula. Dis Colon Rectum.

